# Shape Information Mediating Basic- and Subordinate-Level Object Recognition Revealed by Analyses of Eye Movements

**DOI:** 10.1037/a0034983

**Published:** 2013-12-23

**Authors:** Lina I. Davitt, Filipe Cristino, Alan C.-N. Wong, E. Charles Leek

**Affiliations:** 1 Wales Institute for Cognitive Neuroscience, School of Psychology, Bangor University, Bangor, UK; 2 Department of Psychology, The Chinese University of Hong Kong, Shatin, New Territories, Hong Kong; 3 Wales Institute for Cognitive Neuroscience, School of Psychology, Bangor University, UK

**Keywords:** eye movements, object recognition, basic-level, subordinate-level, image classification

## Abstract

This study examines the kinds of shape features that mediate basic- and subordinate-level object recognition. Observers were trained to categorize sets of novel objects at either a basic (between-families) or subordinate (within-family) level of classification. We analyzed the spatial distributions of fixations and compared them to model distributions of different curvature polarity (regions of convex or concave bounding contour), as well as internal part boundaries. The results showed a robust preference for fixation at part boundaries and for concave over convex regions of bounding contour, during both basic- and subordinate-level classification. In contrast, mean saccade amplitudes were shorter during basic- than subordinate-level classification. These findings challenge models of recognition that do not posit any special functional status to part boundaries or curvature polarity. We argue that both basic- and subordinate-level classification are mediated by object representations. These representations make explicit internal part boundaries, and distinguish concave and convex regions of bounding contour. The classification task constrains how shape information in these representations is used, consistent with the hypothesis that both parts-based, and image-based, operations support object recognition in human vision.

Our ability to recognize familiar objects across variations in sensory input arising from changes in scale, viewpoint, lighting, and other factors is one of the most remarkable aspects of human vision (e.g., Arguin & Leek, 2003; Biederman, 1987; Bukach, Gauthier, & Tarr, 2006; Foster & Gilson, 2002; Harris, Dux, Benito, & Leek, 2008; Leek, 1998a, 1998b; Leek, Atherton, & Thierry, 2007; Leek & Johnston, 2006; Wong & Hayward, 2005). We are also adept at recognizing objects at different levels of classification. For example, we can rapidly classify unfamiliar vehicles into known basic-level categories such as “car,” “truck,” and “bus.” Using prior knowledge, and expertise, we can also individuate exemplars within basic-level categories at subordinate levels (e.g., “Renault 5,” “Seat Ibiza,” and “BMW X3”). Despite the apparent ease with which observers accomplish object recognition we know relatively little about how the visual system represents shape, and what image features are used to support different levels of shape classification.

We investigated this issue using analyses of fixational eye movement patterns. Leek et al. (2012) found that the spatial distributions of fixation patterns during the perception of complex surface-rendered novel objects was strongly influenced by surface curvature polarity. Observers showed a preference for fixation at regions of concave relative to convex surface intersection. This finding is consistent with those from other studies, largely based on shape judgments about contour-based 2D polygons, that observers show greater sensitivity to changes in the magnitude and sign of curvature at concave than convex regions (e.g., Attneave, 1954; Barenholtz, Cohen, Feldman, & Singh, 2003; Bertamini, 2008; Biederman, 1987; Cate & Behrmann, 2010; Cohen, Barenholtz, Singh, & Feldman, 2005; Cohen & Singh, 2007; De Winter & Wagemans, 2006; Feldman & Singh, 2005; Hoffman & Richards, 1984; Hoffman & Singh, 1997; Lim & Leek, 2012). Formally, it has been demonstrated that, for geometrically closed forms, concave regions carry more shape information (or surprisal) than convex regions (Feldman & Singh, 2005; Lim & Leek, 2012; also see Singh & Feldman, 2012). One influential hypothesis is that curvature polarity on object structure provides a cue to the presence of part boundaries, particularly at concave regions formed by the intersection of object parts (Hoffman & Richards, 1984). This proposal is consistent with structural description models of basic (or entry)-level recognition in which complex objects are represented in terms of their constituent parts and spatial configuration (e.g., Biederman, 1987; Hummel & Stankiewitz, 1996; Leek, Reppa, Rodriguez, & Arguin, 2009; Leek, Reppa, & Arguin, 2005; Marr & Nishihara, 1978). In contrast, the importance of part boundaries and differential sensitivity to curvature polarity in shape perception appears to challenge other models of recognition such as image-based hierarchical accounts like HMAX (e.g., Serre, Oliva, & Poggio, 2007; Serre, Wolf, Bileschi, Riesenhuber, & Poggio, 2007) that do not attribute any special functional status to these kinds of features.

One theoretical possibility is that basic- and subordinate-level classification are mediated by different kinds of shape representations: Basic-level classification is supported by a parts-based structural description, and subordinate-level classification is supported by image-based representations, consistent with a hybrid “dual coding” account (e.g., Foster & Gilson, 2002; Hummel & Stankiewicz, 1996). In this case, we might only expect to find evidence for the differential processing of concave features like part-boundaries during basic-level classification.

To test this we analyzed the spatial distributions of fixational eye movement patterns recorded while observers made either basic- (between-families) or subordinate- (within-family) level classification judgments about sets of surface rendered solid novel objects. Fixation patterns were compared to algorithmically generated shape feature models based on convex and concave regions of bounding contour, and internal part boundaries.

## Methods

### Participants

Twenty-four students from Bangor University participated in exchange for course and printer credits. Twelve were assigned to a subordinate-level classification group (8 females, *M* age = 20.08, *SD* = 2.5), and 12 to a basic-level classification group (9 females, *M* age = 19.75, *SD* = 1.05). All reported normal or corrected to normal vision. The protocol had received approval from the local Research Ethics and Governance Committee.

### Stimuli

**Figure 1 fig1:**
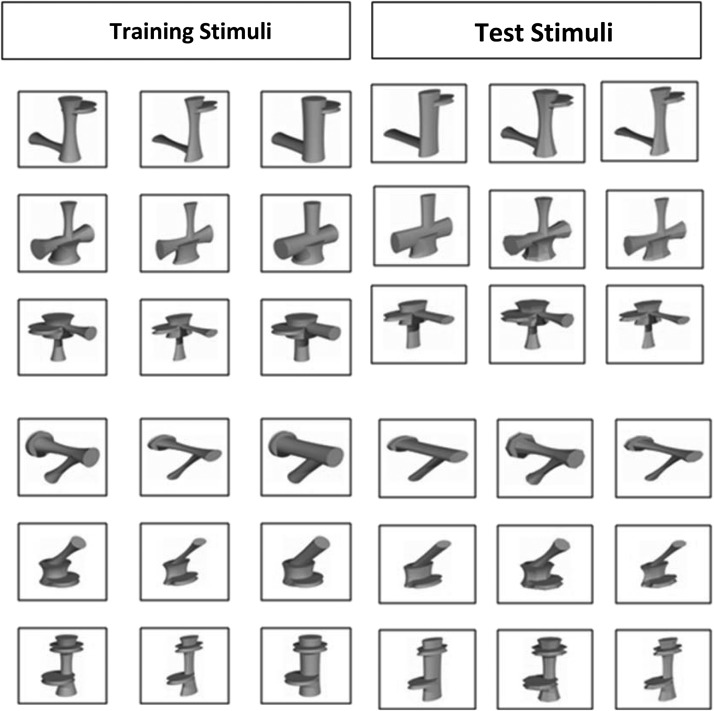
The stimuli set (Ziggerins) used in the experiment.

The stimuli comprised 36 of the novel (“Ziggerins”) objects developed by Wong, Palmeri, & Gauthier (2008; see Figure 1). There were six different classes of Ziggerins each defined by a distinctive part structure. Each class consisted of six Ziggerins defined by distinct metric variation of part size, aspect ratio, and cross-sectional shape. The models were rendered in yellow and scaled to fit within an 800 × 800 pixel frame subtending 18° of visual angle horizontally from the viewing distance of 60 cm.[Fig-anchor fig1]

### Apparatus

Eye movement data were recorded on a Tobii ET-17 binocular eye-tracker (60 Hz sampling). Stimuli were presented on a TFT monitor running at a resolution of 1280 × 1024 pixels and 60 Hz refresh rate.

### Design and Procedure

**Figure 2 fig2:**
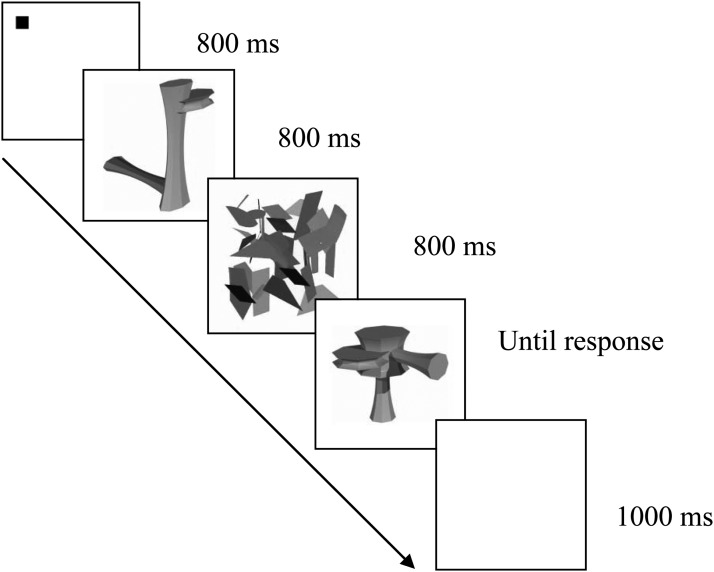
Schematic illustration of the trial structure for the sequential matching task.

There were two tasks: Basic-level versus subordinate-level object classification manipulated as a between-subjects factor. Participants (*N* = 12 per group) were randomly assigned to either the basic- or subordinate-level classification task group and received extensive training according to group assignment following the protocol described by Wong et al. (2008). For each group, this comprised 3 hr (in separate one hour sessions) of tasks requiring either basic- or subordinate-level classification using 18 of 36 randomly selected Ziggerins. The remaining 18 stimuli were used to assess classification performance in a subsequent test phase. Each training session included a sequence of five tasks: inspection, feedback, naming, verification, and matching.^1^ Reaction times and accuracy were recorded during training to monitor participant engagement. The subordinate group learned the individual names for 12 of 18 Ziggerins, with the remaining six objects being used as distracters. The basic group learned to categorize 12 Ziggerins into four different classes, with two unnamed classes (six Ziggerins) used as distracters. An initial random selection of targets and distractor sets was made, but this was used consistently across participants. Two-syllable nonword names given to individual or class objects were randomly assigned (e.g., Rofo, Vilo). After training, basic- or subordinate-level classification performance was assessed using a sequential matching task (*N* trials = 216) following Wong et al. (2008; see Figure 2). For the subordinate-level task, the participants judged (by keyboard response: same/different) whether the two Ziggerins were the same or different individual exemplar. For the basic-level task, participants judged whether the two Ziggerins were from the same or different family.[Fig-anchor fig2]

### Analyses of Eye Movement Data

Eye tracking data analysis parameters were identical to those used by Leek et al. (2012). The first fixation on each trial was discarded. Analyses of the eye movement data were based on the fixation region overlap analysis (FROA) methodology (see Johnston & Leek, 2009; Leek et al., 2012; for more details). FROA allows the spatial distributions of observed fixation patterns to be statistically compared to models of the spatial distributions of image features of interest. The key dependent measure in FROA is known as *model matching correspondence* (MMC) which quantifies the difference in the degree of spatial overlap between the observed distribution of fixations and a given theoretical model (e.g., spatial distribution of concave curvature minima) relative to the 95% confidence interval (CI) of the chance overlap distribution determined by Monte Carlo. Higher values of MMC indicate better data-model correspondences (where negative values indicate percentage overlap that is less than the amount expected at the 95% CI). MMC statistics were subjected to analyses of variance (ANOVA). Eye movement analyses were performed on data from the sequential matching task only.

### Generating Model Predictions

**Figure 3 fig3:**
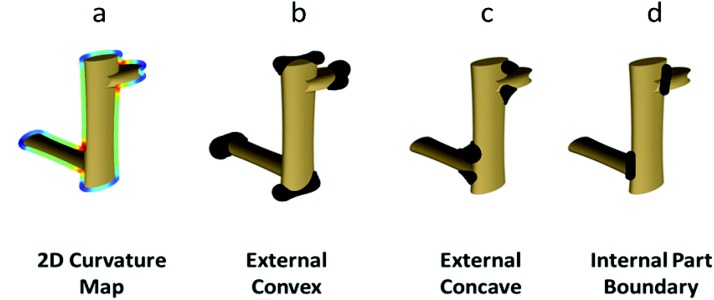
Examples of the algorithmically generated Regions Of Interest (ROIs) for each model: (a) the 2D curvature map used to define (b) external convex and (c) concave regions; and (d) internal part boundaries defined by the minima/short-cut rule (see the Methods section).

Fixation patterns were compared against three algorithmically generated models: concave regions of bounding contour, convex regions of bounding contour, and internal part boundaries (see Figure 3). The bounding curvature models were based on contour-based curvature maps extracted from image silhouettes for each stimulus using the algorithm described in Feldman and Singh (2005). For each discrete point on the silhouette, the curvature sign and magnitude was computed and transformed into a proportionally sized circular disk, which were summed to create the final concave and convex masks. The corresponding masks served as Regions Of Interest (ROIs) in the FROA analysis. The internal part-boundary models were created by defining the intersection between 3D parts using the ‘minima short-cut’ rule between paired concavities (e.g., Singh & Hoffman, 2001; Singh, Seyranian, & Hoffman, 1999).[Fig-anchor fig3]

## Results

### Behavioral Data

Accuracy (% correct) for the basic-level classification task (% correct; *M* = 95%; *SD* = .07) was not significantly different from the subordinate-level classification task (*M* = 96%; *SD* = .02; Mann–Whitney, *p* = .471). For RTs, an independent *t* test (correct responses) showed no significant difference between basic- (*M* = 660.62 ms; *SD* = 114.05) and subordinate-level classification (*M* = 632.35 ms; *SD* = 114.05; *t*(18) = −.622, *p* = .542).

### Analyses of the Spatial Distributions of Fixations

**Figure 4 fig4:**
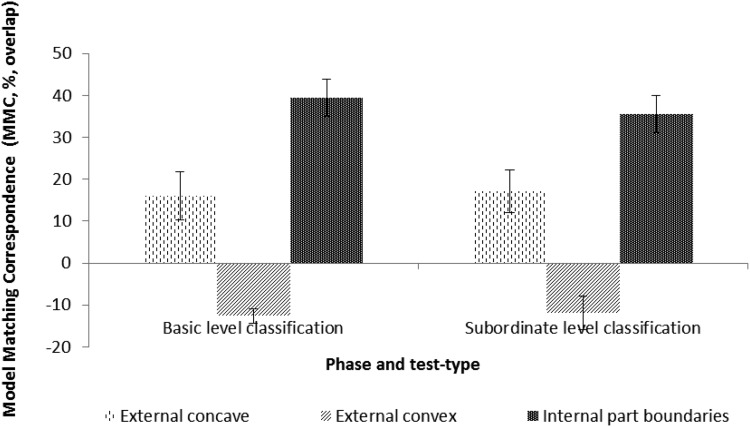
Data-model correspondences across tas*ks.* These are expressed (see the Methods section) in terms of model matching correspondence (MMC). MMC values are expressed relative to the percentage of overlap accounted for by variation in low-level image statistics or visual saliency (see Leek et al., 2012) by generating fixation overlap distributions relative to those predicted by the saliency model. A positive MMC value indicates a higher fixation data-model correspondence than that accounted for by visual saliency. In contrast, a negative value indicates a lower fixation data-model correspondence than that accounted for by visual saliency. Bars show standard error of the mean (% overlap).

The goal of these analyses was to determine whether the patterns of data-model correspondences were modulated by classification task. The mean MMC values for the external concave and convex models, and internal part boundary models (relative to the visual saliency baseline) as a function of classification task (basic vs. subordinate) are shown in Figure 4.[Fig-anchor fig4]

A 2 (Task: Basic, Subordinate) × 3 (Model: External Concave, External Convex, Internal Part Boundaries) mixed ANOVA showed a significant main effect of Model, *F*(2, 34) = 47.92, *p* < .0001, ηp^2^ = .738. There were no other main effects or interactions.

Planned comparisons between models for the basic task were significant for external concave versus external convex, *p* < .0001; external concave versus internal part boundaries, *p* = .011; and external convex versus internal part boundaries, *p* < .0001. Planned comparisons for the subordinate task were significant for external concave versus external convex, *p* < .0001; external concave versus internal part boundaries, *p* = .006; and external convex versus internal part boundaries, *p* = .001. There were no significant differences for models between the basic and the subordinate task.

### Further Analyses

The overall mean saccade amplitude was lower for the basic (*M* = 2.30°; *SD* = 1.26) than subordinate classification task (*M* = 3.64°; *SD* = 1.71), *t*(23) = −5.027, *p* < .0001. There was no difference in mean dwell times (basic: *M* = 198.15 ms; *SD* = 46.21; subordinate: *M* = 195.99 ms; *SD* = 45.36), *t*(23) = .268, *p* = .791.

## General Discussion

The results extend those reported by Leek et al. (2012) by showing fixation preferences for internal part boundaries, and for concave over convex regions of bounding contour both during basic- and subordinate-level object recognition. These findings suggest that both basic- and subordinate-level classification are mediated by object representations that make explicit internal part boundaries, and differentially encode concave and concave regions of bounding contour - consistent with parts-based, structural description, approaches (e.g., Biederman, 1987; Hummel & Stankiewitz, 1996; Leek et al., 2005; Marr & Nishihara, 1978). In contrast, the data challenge models of shape representation that do not attribute functional significance to these image features including recent variants of feed-forward image-based models like HMAX (Serre, Wolf, et al., 2007; Serre, Oliva, & Poggio, 2007). To account for these data, such models would require modification to include a level of feature representation that makes explicit internal part boundaries, and the sign of curvature along bounding contour.

Although the spatial distribution of fixations across these image features was the same for basic- and subordinate-level recognition, the analysis of saccade amplitudes revealed a difference in scanning patterns: Saccade amplitudes were shorter in the basic- than subordinate-level classification task. This provides evidence for differential shape information processing. To understand this pattern of results, it is useful to consider task requirements. Subordinate-level classification could only be performed by a perceptual analysis of metric differences in global shape properties (see Figure 1). Because stimuli within an object class had the same part configuration, local part relations might be expected to place weaker constraints on image processing—equivalent to the perceptual processing demands of subordinate-level classification among common objects (where all exemplars share the same overall part configuration). As a consequence, shape information may need to be sampled more globally across object structure—correlating with higher saccade amplitudes. In contrast, basic-level discrimination could be constrained more strongly by computing local (internal) part relations, and their corresponding structural descriptions, which would be sufficient for distinguishing among members of each object class. Thus, although the representations mediating recognition may be common to basic- and subordinate-level classification—as suggested by the similarity of correspondences between fixation patterns and feature models across tasks—the way information in those representations is sampled may be differentially constrained by task demands.

Finally, it is interesting to consider the role of concave regions in image classification. As we have noted, a key hypothesis is that these regions fundamentally underpin perceptual analyses of object part boundaries and compositional structure. However, this does not necessarily mean that concave regions constrain basic- and subordinate-level classification in the same way. Concave regions may not only derive functional significance as part boundaries, but also as local features, keypoints or interest point operators that constrain generalization across views—as in, for example, Ullman (1996). Thus, these image features may support the computation of parts-based structural description representations during basic-level classification and serve as local geometric keypoints to constrain image-based processing during subordinate-level classification. This would be consistent with the possibility that both parts-based, and image-based, operations support object recognition (e.g., Foster & Gilson, 2002; Hummel & Stankiewitz, 1996).

## Footnotes

1 In the inspection task, a Ziggerin and verbal label (name or family) were shown together with no response requirement. In the feedback task, a single Ziggerin was shown and the first letter of its name/family had to be indicated. Correct/incorrect feedback was given. The naming task was identical except no feedback was provided. During the verification task, a single name/family was shown followed by a Ziggerin that remained on the screen until a match/mismatch response was made. In the matching task, a name/family was presented followed two Ziggerins presented side by side. Participants indicated which Ziggerin matched the verbal label.
